# Supplementary material for: Active site specificity profiling datasets of matrix metalloproteinases (MMPs) 1, 2, 3, 7, 8, 9, 12, 13 and 14
Source: Data Brief. 2016 Feb 22;7:299–310. doi: 10.1016/j.dib.2016.02.036 (PMC4777984; doi:10.1016/j.dib.2016.02.036)
Supplement: Supplementary file 10 — Supplementary material [file mmc10.zip › WebPICS_hMMP3_T_1%/subsite_coop.html]

 

PICS results


- # About
- # Manual
- # Analysis
- # Results

- Redraw
- Seqlogo
- Dependency
- Coop

**Potential subsite cooperativity analysis for hMMP3\_T\_1%**   
Minimum difference for subsite dependency was set to +- 10 percentage-points  
Subsite dependency was checked for all positional occurences above 1 x natural abundance  
**Subsite dependency check is restricted to P3 - P3'**

|  |  |  |  |
| --- | --- | --- | --- |
| Fixed residue | Affected residue(s) | Change (percentage-points) | Vice-Versa change |
| P3\_A | P1\_N | 12.9 | 12.9 |
| P3\_A | P1prime\_I | 18.2 | 14.6 |
| P3\_A | P3prime\_Q | 10.7 | 21.2 |
| P3\_C | P2prime\_I | 88.9 | 12.1 |
| P3\_H | P1\_Q | 27.8 | 15.2 |
| P3\_H | P2\_L | 39.4 | 11.3 |
| P3\_H | P2\_W | 14.7 | 22.0 |
| P3\_H | P2prime\_Q | 27.8 | 15.2 |
| P3\_I | P1\_D | 18.8 | 15.3 |
| P3\_I | P1prime\_C | 12.6 | 21.5 |
| P3\_I | P3prime\_A | 22.7 | 15.5 |
| P3\_I | P3prime\_E | 10.6 | 12.9 |
| P3\_L | P1\_E | 32.1 | 18.4 |
| P3\_L | P2prime\_T | 11.6 | 13.4 |
| P3\_L | P3prime\_Q | 11.4 | 21.7 |
| P3\_V | P1\_G | 22.8 | 27.8 |
| P3\_V | P1\_S | 10.2 | 13.9 |
| P3\_V | P2\_G | 14.7 | 12.9 |
| P2\_F | P1\_P | 24.3 | 12.2 |
| P2\_F | P3prime\_H | 19.2 | 28.8 |
| P2\_M | P1\_F | 38.9 | 34.0 |
| P2\_M | P2prime\_H | 11.3 | 13.2 |
| P2\_M | P2prime\_M | 39.4 | 39.4 |
| P2\_M | P3prime\_G | 56.3 | 13.2 |
| P2\_Q | P1\_S | 12.0 | 11.3 |
| P2\_Q | P1prime\_M | 10.3 | 25.8 |
| P2\_Q | P2prime\_I | 15.6 | 10.7 |
| P2\_Q | P3prime\_C | 11.8 | 59.2 |
| P2\_S | P1\_S | 27.7 | 24.2 |
| P2\_S | P1prime\_W | 11.3 | 26.3 |
| P2\_S | P3prime\_Q | 15.4 | 18.0 |
| P2\_W | P1prime\_F | 21.0 | 10.5 |
| P1\_A | P3prime\_A | 13.5 | 12.4 |
| P1\_D | P1prime\_F | 10.3 | 26.9 |
| P1\_F | P1prime\_V | 46.9 | 12.1 |
| P1\_F | P2prime\_M | 34.0 | 38.9 |
| P1\_G | P1prime\_Q | 11.2 | 18.3 |
| P1\_G | P1prime\_W | 19.2 | 57.7 |
| P1\_N | P2prime\_V | 16.1 | 14.8 |
| P1\_P | P1prime\_V | 17.7 | 10.4 |
| P1\_P | P3prime\_H | 19.2 | 57.7 |
| P1\_Q | P2prime\_I | 25.3 | 12.7 |
| P1\_Q | P3prime\_T | 29.4 | 23.1 |
| P1\_S | P3prime\_C | 11.0 | 58.7 |
| P1\_S | P3prime\_E | 18.0 | 20.6 |
| P1prime\_F | P3prime\_T | 18.0 | 10.3 |
| P1prime\_M | P2prime\_H | 13.7 | 13.7 |
| P1prime\_M | P3prime\_T | 26.3 | 11.3 |
| P1prime\_Q | P2prime\_Q | 30.9 | 30.9 |
| P1prime\_Q | P3prime\_H | 15.2 | 27.8 |
| P1prime\_Q | P3prime\_Q | 21.3 | 19.5 |
| P1prime\_V | P2prime\_I | 11.5 | 16.2 |
| P1prime\_V | P3prime\_G | 10.7 | 11.1 |
| P1prime\_W | P2prime\_M | 13.2 | 11.3 |
| P1prime\_W | P3prime\_H | 13.7 | 13.7 |
| P2prime\_Q | P3prime\_H | 15.2 | 27.8 |
| P2prime\_Q | P3prime\_Q | 12.2 | 11.2 |

  
**PICS analysis of protease: hMMP3\_T\_1%**  
199 cleavage sites analyzed  
PICS library made with (T)rypsin, (G)luC or (C)hymotrypsin:   
Cutoff for graphic display: 2 x natural abundance  

|  |  |
| --- | --- |
| Positional occurences  (table for total and relative (in %) values) | Occurences relative to natural abundance (table) |
|  |  |
